# Non-Alcoholic Fatty Liver Disease and Hypokalemia in Primary Aldosteronism Among Chinese Population

**DOI:** 10.3389/fendo.2021.565714

**Published:** 2021-04-22

**Authors:** Yi Chen, Xueyang Chen, Qiang Chen, Chaohui Yu

**Affiliations:** Department of Gastroenterology, The First Affiliated Hospital, College of Medicine, Zhejiang University, Hangzhou, China

**Keywords:** hypokalemia, non-alcoholic fatty liver disease, primary aldosteronism, inflammation, insulin resistance

## Abstract

**Background:**

In recent years, evidence that aldosteronism is a risk factor for metabolic disorders has increased. This study was designed to investigate the role of nonalcoholic fatty liver disease (NAFLD) and hypokalemia in primary aldosteronism (PA).

**Methods:**

A total of 222 patients diagnosed with PA and 222 non-PA patients were included in our study. Demographic data, medical histories, clinical evaluations, complete blood counts, serum biochemical analyses, aldosterone and potassium levels were obtained. Data are presented as the means ± standard deviation (SD). To compare the parameters between cases and controls, Student’s t-tests or Mann-Whitney U tests were used for continuous variables, and χ2 tests were used for categorical variables. Pearson correlation analysis was used to define relationships between pairs of parameters. A two-sided *P* < 0.05 was considered statistically significant. Multivariate logistic regression was performed to assess the independent effects of potassium and other metabolic variables on NAFLD in PA patients.

**Results:**

The diagnosis of NAFLD was more common in PA patients (n=222, 35.1%) than in non-PA subjects (29.7%). PA patients with and without NAFLD had similar metabolic imbalance characteristics. In PA patients with hypokalemia, relatively higher prevalences of NAFLD (44% vs. 27%, *P* < 0.05) and diabetes mellitus (19.8% vs. 9.9%, *P* < 0.05) were observed. Hypokalemic PA patients had a worse metabolic status than PA patients without hypokalemia, including higher body mass index (BMI) (25.4 ± 3.4 vs. 24.1 ± 3.9 kg/m^2^, *P* < 0.05), more severe dyslipidemia as well as insulin resistance, higher serum uric acid levels (354 ± 95 vs. 319 ± 87 μmol/L, *P* < 0.01) and aggravated inflammation.

**Conclusion:**

The prevalence of NAFLD was higher in PA patients than in non-PA patients, although the patterns of obesity, dyslipidemia and insulin resistance were similar. Hypokalemic PA patients had a worse metabolic status than normokalemic PA patients. This study provides new insights that can inform further mechanistic studies about metabolic imbalance in patients with aldosteronism.

## Highlights

*The prevalence of NAFLD was higher in primary aldosteronism patients than in people without aldosteronism.
*NAFLD patients with and without primary aldosteronism showed similar patterns of obesity, dyslipidemia and insulin resistance.
*Hypokalemia was accompanied by a worse metabolic status and exacerbated inflammation in patients with primary aldosteronism.
*This study provided new insights that can inform further mechanistic studies about metabolic imbalances in primary aldosteronism patients.

## Introduction

Primary hyperaldosteronism (PA), which was first described by Jerome Conn in the 1950s, is characterized by inappropriate endogenous production of the mineralocorticoid aldosterone by one or both adrenal glands. Recent studies have shown that excess aldosterone is associated with metabolic disorders, such as diabetes (1, 2). Disrupted renin-angiotensin-aldosterone system (RAAS) activity and hypokalemia are fundamental aspects of PA; furthermore, aldosterone-induced hypokalemia has been reported to impair glucose tolerance by impeding insulin secretion (3). Retrospective analyses have suggested that interrupting the RAAS with angiotensin-converting enzyme inhibitors (ACEIs) or angiotensin receptor blockers (ARBs) prevents the occurrence of metabolic disorders, exerting beneficial effects on glucose metabolism and insulin sensitivity (4, 5).

The prevalences of metabolic diseases, including obesity, nonalcoholic fatty liver disease (NAFLD), and diabetes, have increased rapidly in recent years (6–8). NAFLD is defined as a clinicopathological syndrome characterized by lipid accumulation in hepatocytes and is commonly accompanied by obesity, dyslipidemia, diabetes, and cardiac and cerebral vascular diseases, accounting for half of chronic liver disease in China (6, 9). The understanding of NAFLD pathogenesis has progressed from the classic ‘two-hit’ model to the current ‘multiple-hit” model that takes into consideration more genetic and environmental factors (10–12). Particular attention has been given to the application of RAAS inhibition to the treatment of NAFLD. RAAS inhibition was reported to affect NAFLD by reducing hepatic inflammation and fibrosis *via* fibroblast inactivity as well as hepatic stellate cell proliferation prevention, leading to improved liver histology and transaminase levels (13, 14).

Therefore, we conducted a case-control study in a Chinese population to describe the metabolic aspects of PA, investigate the prevalence and metabolic characteristics of NAFLD in PA patients and determine the relationship between hypokalemia and NAFLD in PA patients.

## Materials and Methods

### Patients

A total of 222 patients diagnosed with PA and 222 non-PA subjects who underwent annual health examinations at the First Affiliated Hospital of Zhejiang University from Jan 2009 to June 2018 were included in our study. The following exclusion criteria were applied (15–17): (1) alcohol consumption greater than 140 g/week for men and 70 g/week for women; (2) history of viral hepatitis, autoimmune hepatitis, or other forms of chronic liver disease; (3) self-reported acute infection within 2 weeks; and (4) body mass index (BMI) less than 18.0 kg/m^2^. The study protocol was approved by the Hospital Ethics Committee of the Affiliated Hospital, Medical School, Zhejiang University, and performed in accordance with the principles of the Declaration of Helsinki. All subjects gave informed consent before enrollment.

### Clinical Evaluation

Blood samples for biochemical and endocrine-metabolic profiles (including total bilirubin, triglycerides, total cholesterol, high-density lipoprotein cholesterol, low-density lipoprotein cholesterol, very-low-density lipoprotein cholesterol, electrolytes, liver enzymes, fasting plasma glucose, Hb1Ac, serum uric acid, etc.) were obtained at 0800 h after overnight fasting upon the patient’s first visit to hospital and at discharge or preparation for surgery. Tests were performed using a Hitachi 7600 autoanalyzer (Hitachi, Tokyo, Japan) (16). For the aldosterone measurements, subjects stood upright for 45 minutes, after which blood samples were collected and assayed (18). Demographic data, medical histories, and health habits were recorded by trained physicians. Standing height and body weight without shoes and with light clothes were measured according to standard procedures. BMI was calculated as body weight (kg) divided by the square of height (meter). Systolic blood pressure (SBP) and diastolic blood pressure (DBP) were measured with a sphygmomanometer in the sitting position. Insulin resistance was calculated using the homeostatic model assessment [HOMA-IR = (fasting glucose/mg*dL^-1^×fasting insulin/mU*L^-1^/405].

### Diagnostic Criteria for NAFLD and Metabolic Syndrome

NAFLD was diagnosed according to the guidelines published by the Chinese Liver Disease Association (6). NAFLD was determined by hepatic ultrasound examination following the exclusion of excessive alcohol consumption and viral or autoimmune liver disease. Hepatic ultrasound examinations were carried out by trained ultrasonographers using a Toshiba Nemio 20 sonography machine with a 3.5-MHz probe (Toshiba, Tokyo, Japan) (16). A third expert consulted in cases of disagreement between the two ultrasonographers.

### Diagnosis of PA

PA was diagnosed according to the 2016 Endocrine Society Clinical Practice Guidelines and a previous study (19, 20). Screening for PA was based on a cutoff value of the upright plasma aldosterone (ng/dl)/plasma renin activity (PRA) (ng/ml/h) ratio > 40 in the presence of an aldosterone level >15 ng/dl and suppressed PRA. Then, a confirmatory saline infusion test (0.9% NaCl 500 ml/h for 4 h) was performed in those with an aldosterone/PRA ratio > 40, and only those with plasma aldosterone levels that failed to fall to <5 ng/dl after the saline infusion were diagnosed with PA. Hypokalemia was defined as a serum potassium concentration less than 3.50 mmol/L. Treatment for hypokalemia included the administration of i.v. potassium chloride or p.o. potassium citrate until the serum potassium level reached the normal range.

### Statistical Analysis

The normality of the distribution of the data was tested with the Kolmogorov-Smirnov test. Normally distributed variables are presented as the means ± standard deviation (SD); variables with skewed distributions are presented as the medians (interquartile ranges). To compare the parameters between cases and controls, Student’s *t*-test or the Mann-Whitney *U* test was performed for continuous variables, and the χ^2^ test was performed for categorical variables. Pearson correlation analysis was used to define the relationship between two parameters. Multivariate logistic regression was performed to assess the independent effects of potassium and other metabolic variables on NAFLD in PA patients. Statistical analyses were performed using SPSS 20.0 (SPSS Inc., Chicago, IL, USA). A two-sided *P* < 0.05 was considered statistically significant.

## Results

### General Information

A total of 222 patients with PA and 222 non-PA counterparts were enrolled in this study. Baseline characteristics are shown in **Supplementary Table 1**. The diagnosis of NAFLD was more common in PA patients (n=78/222, 35.1%) than in non-PA patients (n=66/222, 29.7%). Furthermore, a lower serum potassium level (3.44 ± 0.63 vs. 3.95 ± 0.33 mmol/L, *P* < 0.05) and higher blood pressure (SBP 144 ± 20 vs. 123 ± 15 mmHg, *P* < 0.05 and DBP 90 ± 14 vs. 76 ± 10 mmHg, *P* < 0.01) were observed in PA patients.

### Metabolic Characteristics of PA and NAFLD

To further analyze the metabolic characteristics of PA and NAFLD, both the PA and non-PA groups were divided into NAFLD and non-NAFLD subgroups, yielding the following subgroups: NAFLD PA, non-NAFLD PA, NAFLD non-PA and non-NAFLD non-PA. Similar metabolic imbalances were seen in the PA NAFLD and non-PA NAFLD subgroups, indicating similar patterns of NAFLD in both PA and non-PA subjects, including a higher BMI (26.8 ± 3.6 vs. 23.5 ± 3.2 kg/m^2^, *P* < 0.001); dyslipidemia with higher levels of total bilirubin (13.2 ± 7.2 vs. 11.3 ± 5.1μmol/L, *P* < 0.05), triglycerides (2.01 ± 0.86 vs. 1.50 ± 0.90 mmol/L, *P* < 0.001), total cholesterol (4.45 ± 0.74 vs. 4.20 ± 0.87 mmol/L, *P* < 0.05), very-low-density lipoprotein cholesterol (VLDL cholesterol) (0.98 ± 0.37 vs. 0.79 ± 0.50 mmol/L, *P* < 0.05) and a lower level of high-density lipoprotein cholesterol (HDL cholesterol) (1.00 ± 0.26 vs. 1.12 ± 0.29 mmol/L, *P* < 0.01); and insulin resistance, indicated by an elevated fasting plasma glucose level (5.44 ± 1.41 vs. 4.83 ± 1.40 mmol/L, *P* < 0.01), Hb1Ac level (6.37 ± 1.20 vs. 5.96 ± 1.34%, *P* < 0.01), and HOMA-IR score (2.76 ± 2.45 vs. 1.47 ± 1.05, *P* < 0.001) (**Table 1**). Elevated liver enzyme levels, including alanine aminotransferase (ALT) (31 ± 21 vs. 19 ± 15 U/L, *P* < 0.001), aspartate aminotransferase (AST) (26 ± 15 vs. 20 ± 13 U/L, *P* < 0.01) and gamma-glutamyl transpeptidase (GGT) (50 ± 45 vs. 26 ± 24 U/L, *P* < 0.05), were also considered markers of liver damage (**Table 1**).

**Table 1:** Metabolic Characteristics of PA and NAFLD.

Variables	PA			Non-PA			*P* ^*^
	NAFLD	Non-NAFLD	*P *	NAFLD	Non-NAFLD	*P *	
n (male/female)	55/23	72/72	< 0.01	46/20	49/107	< 0.05	0.916
Age (year)	50±11	52±11	0.229	51±12	52±12	0.326	0.934
Body mass index (kg/m^2^)	26.8±3.6	23.5±3.2	< 0.001	26.4±3.4	22.1±2.8	< 0.001	0.645
Systolic blood pressure	146±20	142±20	0.274	130±14	115±16	< 0.001	< 0.05
Diastolic blood pressure	91±14	89±14	0.188	78±10	72±10	< 0.001	< 0.05
ALT (U/L)	31±21	19±15	< 0.001	30±19	18±13	< 0.001	0.897
AST (U/L)	26±15	20±13	< 0.01	23±8	19±6	< 0.001	0.564
ALP (U/L)	74±22	71±22	0.388	74±19	69±18	0.256	0.976
GGT (U/L)	50±45	26±24	< 0.05	45±39	23±17	< 0.001	0.526
Total bilirubin (μmol/L)	13.2±7.2	11.3±5.1	< 0.05	13.7±5.4	12.4±4.7	0.090	0.946
Triglyceride (mmol/L)	2.01±0.86	1.50±0.90	< 0.001	2.00±1.23	1.06±0.55	< 0.001	0.848
Total cholesterol (mmol/L)	4.45±0.74	4.20±0.87	< 0.05	4.95±0.95	4.50±0.84	< 0.001	0.286
HDL-cholesterol (mmol/L)	1.00±0.26	1.12±0.29	< 0.01	1.19±0.28	1.42±0.35	< 0.001	0.497
LDL-cholesterol (mmol/L)	2.48±0.58	2.33±0.73	0.102	2.84±0.81	1.41±0.65	< 0.001	0.376
VLDL-cholesterol (mmol/L)	0.98±0.37	0.79±0.50	< 0.05	0.94±0.39	0.77±0.48	< 0.05	0.536
Fasting plasma glucose (mmol/L)	5.44±1.41	4.83±1.40	< 0.01	5.08±0.83	4.73±0.58	< 0.001	0.364
Hb1Ac (%)	6.37±1.20	5.96±1.34	< 0.01	6.35±1.21	5.90±1.24	< 0.05	0.849
HOMA-IR	2.76±2.45	1.47±1.05	< 0.001	2.56±1.31	1.31±1.08	< 0.001	0.476
Serum uric acid (μmol/L)	356±85	326±95	< 0.05	376±84	316±81	< 0.001	0.485
CRP (mg/L)	3.16±2.20	2.45±2.11	0.060	3.25±2.10	2.65±1.91	0.151	0.863
Aldosterone (pg/mL)	321±201	238±161	0.173				
Potassium (mmol/L)	3.37±0.61	3.48±0.63	0.201				
PRA (ng/mL/h)	0.74±0.56	0.38±0.23	0.071				
Ang II (pg/mL)	7.3±3.8	7.2±3.5	0.924				
Urinary aldosterone (μg/24 h)	9.0±6.1	6.3±4.0	< 0.01				
Urine potassium (mmol/24 h)	46.9±20.9	49.2±38.4	0.641				
Metabolic Comorbidities (n/%)							
Diabetes mellitus	15/19%	18/13%	0.159				
Coronary artery disease	2/2.6%	1/0.7%	0.204				
Carotid atherosclerosis	15/19.2%	17/15.9%	0.335				

HDL-cholesterol, high-density-lipoprotein cholesterol; LDL-cholesterol, low-density-lipoprotein cholesterol; VLDL-cholesterol, very-low-density-lipoprotein cholesterol; CRP, C-reactive protein; ALT, alanine aminotransferase; AST, aspartate aminotransferase; ALP, alkaline phosphatase; GGT, gamma-glutamyl transpeptidase; PRA, plasma renin activity. ^*^P compare variables between NAFLD in PA patients and NAFLD in non-PA group.

Further comparing NAFLD in PA and non-PA subjects, the metabolic characteristics were similar, except the PA NAFLD subgroup had higher blood pressure values than the non-PA NAFLD subgroup (SBP 146 ± 20 mmHg vs. 130 ± 14 mmHg, *P* < 0.05 and DBP 91 ± 14 vs. 78 ± 10 mmHg, *P* < 0.05).

### Metabolic Characteristics in Hypokalemic PA

Given the similarities in the PA NAFLD and non-PA NAFLD subgroups, we specifically investigated the PA group (n=222) to determine if metabolic changes were present in those with hypokalemia compared with those with normal potassium levels. The hypokalemia subgroup had higher prevalences of NAFLD (44% vs. 27%, *P* < 0.05) and diabetes mellitus (19.8% vs. 9.9%, *P* < 0.05) (**Table 2** and **Supplementary Figure 1**). Patients with hypokalemia had a worse metabolic status, with a higher BMI (25.4 ± 3.4 vs. 24.1 ± 3.9 kg/m^2^, *P* < 0.05); worse dyslipidemia with higher total bilirubin (13.0 ± 7.0 vs. 10.9 ± 4.6 μmol/L, *P* < 0.05), triglyceride (1.88 ± 0.83 vs. 1.48 ± 0.96 mmol/L, *P* < 0.01), total cholesterol (4.42 ± 0.84 vs. 4.16 ± 0.82 mmol/L, *P*< 0.05), LDL cholesterol (2.48 ± 0.68 vs. 2.28 ± 0.68 mmol/L, *P* < 0.05), and VLDL cholesterol (0.93 ± 0.38 vs. 0.78 ± 0.54 mmol/L, *P* < 0.05) levels and a lower HDL cholesterol level (1.00 ± 0.27 vs. 1.14 ± 0.28 mmol/L, *P* < 0.001); and worse insulin resistance (HOMA-IR: 2.30 ± 2.34 vs. 1.56 ± 0.65, *P* < 0.001). Furthermore, the hypokalemia subgroup had a significantly elevated serum uric acid level (354 ± 95 vs. 319 ± 87 μmol/L, *P* < 0.01) (**Table 2**). In the Pearson correlation analysis, serum potassium was weakly positively related to HDL cholesterol (β = 0.195, *P*< 0.01) and weakly negatively related to BMI (β = -0.149, *P*< 0.05), total bilirubin (β = -0.189, *P*< 0.01), and LDL cholesterol (β = -0.139, *P*< 0.05) (**Supplementary Figure 2**).

**Table 2:** Metabolic characteristics in normokalemia and hypokalemia patients with primary aldosteronism.

Variables	Normokalemia	Hypokalemia	*P *
n	111	111	
Aldosterone (pg/mL)	229±127	323±217	< 0.05
Potassium (mmol/L)	3.94±0.18	2.95±0.38	< 0.001
Potassium supplementation	2/2%	105/95%	< 0.001
PRA (ng/mL/h)	0.473±0.173	0.209±0.115	< 0.05
Urinary aldosterone (μg/24 h)	6.33±4.04	8.96±6.11	< 0.05
Urine potassium (mmol/24 h)	45.5±28.6	50.3±20.7	< 0.05
Body mass index (kg/m^2^)	24.1±3.9	25.4±3.4	< 0.05
Systolic blood pressure	143±20	145±21	0.647
Diastolic blood pressure	88±14	91±14	0.165
Total bilirubin (μmol/L)	10.9±4.6	13.0±7.0	< 0.05
Triglyceride (mmol/L)	1.48±0.96	1.88±0.83	< 0.01
Total cholesterol (mmol/L)	4.16±0.82	4.42±0.84	< 0.05
HDL-cholesterol (mmol/L)	1.14±0.28	1.00±0.27	< 0.001
LDL-cholesterol (mmol/L)	2.28±0.68	2.48±0.68	< 0.05
VLDL-cholesterol (mmol/L)	0.78±0.54	0.93±0.38	< 0.05
Fasting plasma glucose (mmol/L)	4.86±1.08	5.21±1.68	0.069
Hb1Ac (%)	6.12±1.34	6.13±1.28	0.964
HOMA-IR	1.56±0.65	2.30±2.04	< 0.05
Serum uric acid (μmol/L)	319±87	354±95	< 0.01
CRP (mg/L)	2.3±1.5	3.1±2.6	< 0.05
Metabolic Comorbidities (n/%)			
NAFLD	30/27%	48/44%	< 0.05
Diabetes mellitus	11/9.9%	22/19.8%	< 0.05
Coronary artery disease	1/0.9%	2/1.8%	0.563
Carotid atherosclerosis	18/16%	14/13%	0.447

HDL-cholesterol, high-density-lipoprotein cholesterol; LDL-cholesterol, low-density-lipoprotein cholesterol; VLDL-cholesterol, very-low-density-lipoprotein cholesterol; CRP, C-reactive protein; PRA, plasma renin activity; NAFLD, non-alcoholic fatty liver disease.

Multivariate logistic regression showed that serum potassium (OR, 5% CI, 1.426, 1.120-1.916, *P* < 0.05), ALT (OR, 5% CI, 1.324, 1.132-1.656, *P* < 0.05) and triglycerides (OR, 5% CI, 1.476, 1.018-2.174, *P* < 0.05) were significantly and independently associated with NAFLD with PA, while BMI, SBP, DBP, HDL cholesterol and fasting plasma glucose were not (**Table 3**).

**Table 3:** Multivariate analysis to assess serum potassium and other metabolic syndrome components in predicting NAFLD among PA.

Variables	OR	95% CI	*P *
Potassium (mmol/L)	1.426	1.120-1.916	< 0.05
Body mass index (kg/m^2^)	1.132	0.875-1.895	0.457
Systolic blood pressure	0.997	0.977-1.019	0.807
Diastolic blood pressure	1.014	0.984-1.058	0.379
ALT (U/L)	1.324	1.132-1.656	< 0.05
Triglyceride (mmol/L)	1.476	1.018-2.174	< 0.05
HDL-cholesterol (mmol/L)	0.389	0.121-1.424	0.169
Fasting plasma glucose (mmol/L)	1.030	0.610-1.184	0.905

HDL-cholesterol, high-density-lipoprotein cholesterol.

### Inflammation in Hypokalemic PA

To further explore the underlying factors influencing the pathogenesis of NAFLD in hypokalemic PA patients, serum inflammatory markers were evaluated. The white blood cell (WBC) count (5.8 ± 1.6 vs. 6.5 ± 1.5, *P* < 0.001), neutrophil count (3.6 ± 1.3 vs. 4.0 ± 1.2, *P* < 0.05), C-reactive protein (CRP) level (2.3 ± 1.5 vs. 3.1 ± 2.6, *P* < 0.05) and platelet-to-lymphocyte ratio (PLR) (89 ± 21 vs. 102 ± 41, *P* < 0.05) were all significantly higher in patients with hypokalemia than in those with normokalemia (**Table 4**).

**Table 4:** Inflammatory status in normokalemia and hypokalemia patients with primary aldosteronism.

Variables	Normokalemia	Hypokalemia	*P*
WBC (*10^9)	5.8±1.6	6.5±1.5	< 0.001
Neutrophil (*10^9)	3.6±1.3	4.0±1.2	< 0.05
CRP (mg/L)	2.3±1.5	3.1±2.6	< 0.05
PLR	89±21	102±41	< 0.05

WBC, white blood count; CRP, C-reactive protein; PLR, platelet-to-lymphocyte ratio.

### Metabolic and Inflammatory State Changes Upon Potassium Supplementation in Hypokalemic PA Patients

Upon discharge or switch to surgical treatment, metabolic status was re-evaluated. It turned out that potassium supplementation in patients with hypokalemia improved the overall metabolic and inflammatory statuses, represented by the reversal of the elevations in the levels of total bilirubin (10.7 ± 5.6 vs. 12.0 ± 5.9 μmol/L, *P* = 0.109), triglycerides (1.43 ± 0.87 vs. 1.69 ± 0.76 mmol/L, *P* < 0.05), total cholesterol (4.13 ± 0.75 vs. 4.38 ± 0.80 mmol/L, *P* < 0.05), HDL cholesterol (1.16 ± 0.30 vs. 1.08 ± 0.29 mmol/L, *P* = 0.123), LDL cholesterol (2.25 ± 0.64 vs. 2.37 ± 0.65 mmol/L, *P* = 0.223), VLDL cholesterol (0.77 ± 0.50 vs. 0.84 ± 0.35 mmol/L, *P* = 0.209), HOMA-IR (1.60 ± 1.27 vs. 2.01 ± 1.84, *P* = 0.069), and serum uric acid (317 ± 89 vs. 325 ± 105 μmol/L, *P* = 0.073) (**Supplementary Table 2**). Regarding inflammation, there were no significant abnormalities in the WBC count (5.9 ± 1.8 vs. 6.3 ± 2.2 *10^9, *P* = 0.108), neutrophil count (3.6 ± 1.5 vs. 4.3 ± 2.3 *10^9, *P* = 0.237), CRP level (2.3 ± 1.7 vs. 2.7 ± 2.5 mg/L, *P* = 0.241) and PLR (88 ± 24 vs. 100 ± 45, *P* = 0.058) in the hypokalemic group after potassium supplementation when compared to the normokalemic PA group (**Supplementary Table 2**).

## Discussion

The current study showed that 35.1% of PA patients had comorbid NAFLD, which was significantly higher than the 29.7% (n=66) in the non-PA group and slightly higher than 29.81% (27.78%-31.93%) in previous reports among general population in China (21). NAFLD patients with PA had metabolic disturbances similar to those in non-PA NAFLD patients. Hypokalemic PA patients had higher prevalence of NAFLD and diabetes mellitus, presenting with a worse metabolic status and more aggravated inflammation, which could be partially reversed by potassium supplementation.

PA has attracted substantial attention in recent decades due to its relationship with metabolic disorders, and similar to our findings, several studies have identified adverse metabolic characteristics in patients with PA, including higher prevalence of metabolic syndrome, hyperglycemia and an elevated incidence of cardiovascular events (22). Additionally, a negative correlation between plasma aldosterone and HDL cholesterol levels was reported in essential hypertensive patients with obesity and insulin resistance early on that was not found in later studies (18, 23). After the correction of excess aldosterone, either by surgery in patients with aldosteronoma or medication in those with hyperplasia, significant improvements in the metabolic parameters were observed (24–26). Consistent with this finding, our study also showed that metabolic disorders, especially NAFLD, diabetes and dyslipidemia, were more prevalent in PA patients than in non-PA patients. It would be interesting to find out whether the metabolic imbalance in these patients improved after they receive treatment for PA, whether *via* surgery or medication; a follow-up study is ongoing.

Insulin resistance has long been regarded as an elementary pathological change in patients with metabolic syndrome, especially NAFLD and diabetes (27). Insulin resistance in PA patients has been reported in several previous studies (28, 29). In our study, higher fasting plasma glucose and Hb1Ac levels as well as HOMA-IR scores were identified in PA patients, especially in those with comorbid NAFLD. The altered carbohydrate metabolism in patients with hyperaldosteronism was initially attributed to impaired insulin secretion from pancreatic beta cells due to hypokalemia or to a direct inhibitory effect of corticosteroids (30). Other mechanisms also include the direct activation of mineralocorticoid receptors and promotion of downstream gene expression, such as the insulin receptor gene, after aldosterone passively diffuses through the cell membrane and binds to mineralocorticoid receptors (31).

Hypokalemia has been regarded as a confounder of metabolic disorders induced by excess aldosterone (30). In fact, hypokalemia can worsen insulin resistance and thus potentially lead to fatty liver. Recent studies have proposed that mechanisms regulating insulin sensitivity in PA patients are mainly dependent on the presence of hypokalemia, whereas the direct effect of excess aldosterone seems to be of minor relevance (32, 33). Similarly, our study revealed a worse metabolic pattern in hypokalemic PA patients, and further Pearson correlation analysis demonstrated associations between serum potassium levels and metabolic factors. Interestingly, potassium supplementation partially improved both metabolic and inflammatory statuses in PA patients, further supporting the relationship between hypokalemia and metabolic disturbances in these patients. However, the exact molecular mechanism underlying the relationship between hypokalemia and metabolic disorders needs further study.

Excess aldosterone is known to play an important role in hypertension in patients with metabolic syndrome and is thought to play a central role in insulin resistance and NAFLD. However, the underlying mechanisms remain unclear (34, 35). Recent studies have proposed several potential mechanisms by which excess aldosterone could induce NAFLD. First, RAAS system compromise reduces hepatic steatosis and prevents liver lipid accumulation by lowering serum free fatty acid levels, inhibiting hepatic fatty acid oxidation, altering VLDL cholesterol secretion, and increasing *de novo* lipogenesis (36). Second, excess aldosterone induces NAFLD through mitochondrial dysfunction, which includes disordered fatty acid oxidation, oxidative phosphorylation, mitochondrial DNA replication, antioxidant status, and apoptosis (37). Third, excess aldosterone also induces oxidative stress, which has negative effects on vascular, cardiac and liver tissue (38). Herein, we found that hypokalemia in PA patients was accompanied by a latent inflammatory status, which might also contribute to liver cell injury and, ultimately, NAFLD pathogenesis.

This study has several limitations. First, although the ultrasound-based diagnosis of NAFLD is widely accepted in the clinical setting as a noninvasive cost-effective method of NAFLD screening, with a sensitivity of 89% and specificity of 93%, liver biopsy is still the diagnostic gold standard (39). Further studies should focus on the relationship between PA and the histological severity of NAFLD. Second, due to the cross-sectional nature of this study and the relatively limited sample size with a small effect size, no causal relationship can be concluded based on the current analysis. In addition, there were confounding factors that could have influenced the reliability of the current results. Further prospective, large-sample cohort studies are needed. Third, adrenal vein samples were not available to test whether PA was unilateral.

## Conclusion

The prevalence of NAFLD in PA patients was higher than that in non-PA patients, although the two groups were similar in terms of the patterns of obesity, dyslipidemia and insulin resistance. Hypokalemic PA patients have a worse metabolic status than normokalemic PA patients. This study provided new insights that can inform further mechanistic studies about metabolic imbalances in patients with aldosteronism.

## Data Availability Statement 

The raw data supporting the conclusions of this article will bemade available by the authors, without undue reservation.

## Ethics Statement

The studies involving human participants were reviewed and approved by Hospital Ethics Committee of the Affiliated Hospital, Medical School, Zhejiang University. The patients/participants provided their written informed consent to participate in this study.

## Author Contributions

YC and XC proposed the initial idea and designed the study procedures. YC and QC collected the demographic and clinical data and conducted the statistical analysis. CY provided supervision and consultation during the entire study. All authors contributed to the article and approved the submitted version.

## Funding

This study was supported by the National Key Research and Development Program of China (No. 2017YFC0908900 to CY), the Science Foundation of the Health Bureau of Zhejiang Province (No. 2017183691 to YC), the National Natural Science Foundation of China, (No. 81700504, to YC), and the Zhejiang Province Public Benefit Technology Research Program (No. LGF19H210001, to YC). The funders did not have any role in the study design, data collection and analysis, decisions regarding data release or manuscript preparation.

## Conflict of Interest

The authors declare that the research was conducted in the absence of any commercial or financial relationships that could be construed as a potential conflict of interest.

## Supplementary Material

Supplementary Figure 1 Prevalence of metabolic components and comorbidities in the hypokalemia and normokalemia groups.

Supplementary Figure 2 Pearson correlation analysis of the serum potassium level and metabolic factors.

## Abbreviations

PA, primary aldosteronism; NAFLD, nonalcoholic fatty liver disease; WBC, white blood cell; CRP, C-reactive protein; PLR, platelet-to-lymphocyte ratio; HDL cholesterol, high-density lipoprotein cholesterol; LDL cholesterol, low-density lipoprotein cholesterol; VLDL cholesterol, very-low-density lipoprotein cholesterol; ALT, alanine aminotransferase; AST, aspartate aminotransferase; ALP, alkaline phosphatase; GGT, gamma-glutamyl transpeptidase; BMI, body mass index; RAAS, renin-angiotensin-aldosterone system; ACEIs, angiotensin-converting enzyme inhibitors; ARBs, angiotensin receptor blockers; SBP, systolic blood pressure; DBP, diastolic blood pressure; PRA, plasma renin activity.
