# Supplementary figures and images for: Non-Alcoholic Fatty Liver Disease and Hypokalemia in Primary Aldosteronism Among Chinese Population
Source: Front Endocrinol (Lausanne). 2021 Apr 22;12:565714. doi: 10.3389/fendo.2021.565714 (PMC8101285; doi:10.3389/fendo.2021.565714)

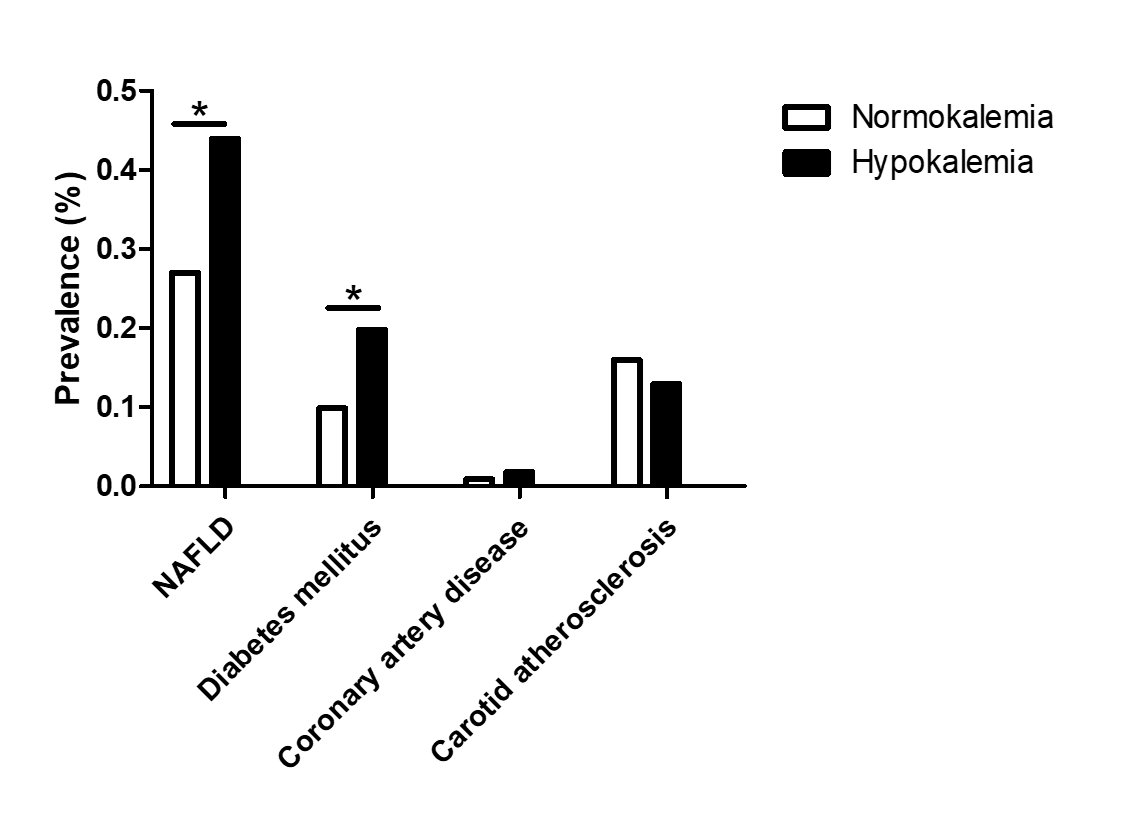

Supplement: Supplementary Figure 1 — Prevalence of metabolic components and comorbidities in the hypokalemia and normokalemia groups. [file Image_1.tif]

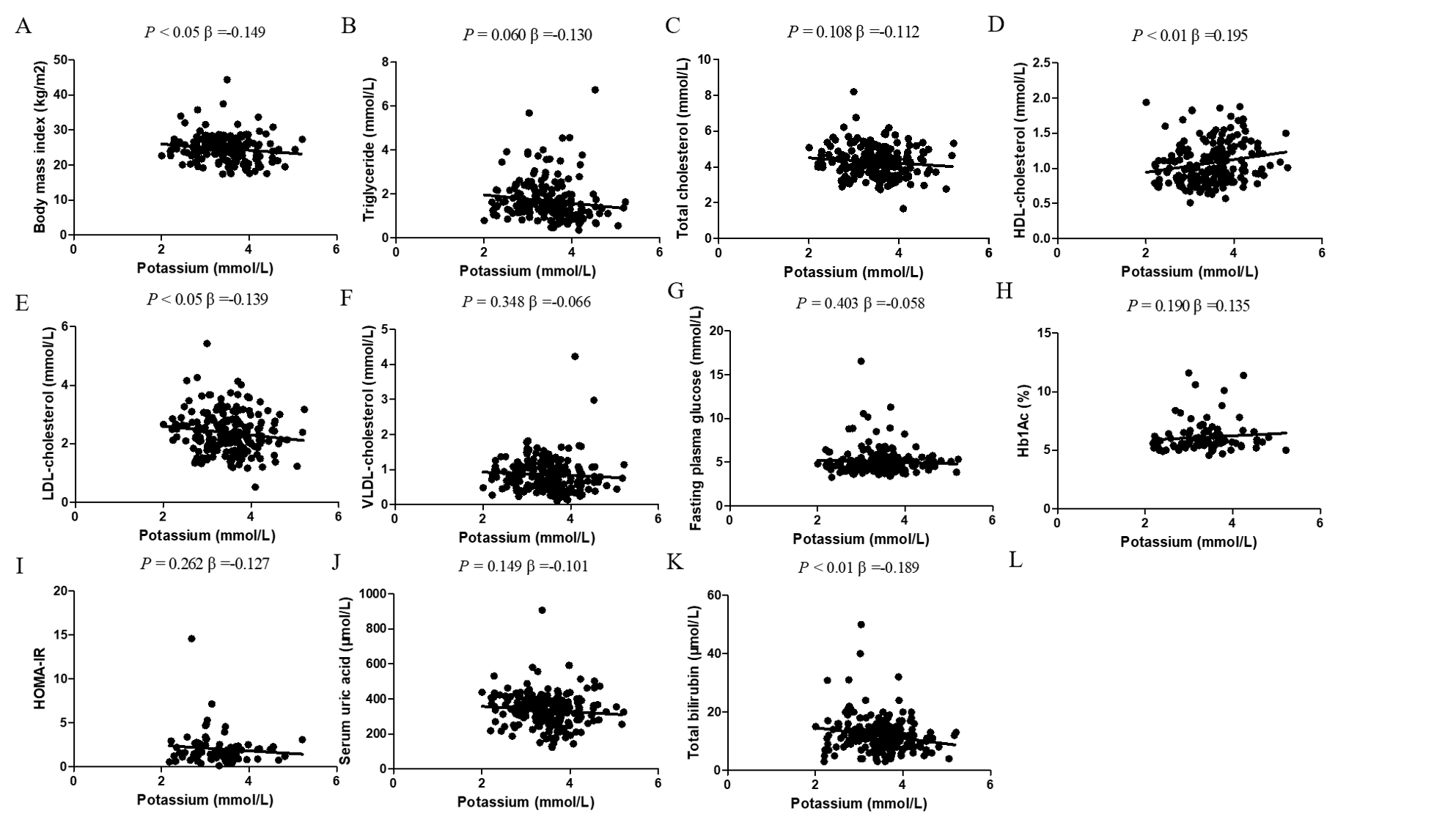

Supplement: Supplementary Figure 2 — Pearson correlation analysis of the serum potassium level and metabolic factors. [file Image_2.tif]
