# Supplementary material for: Non-Alcoholic Fatty Liver Disease and Hypokalemia in Primary Aldosteronism Among Chinese Population
Source: Front Endocrinol (Lausanne). 2021 Apr 22;12:565714. doi: 10.3389/fendo.2021.565714 (PMC8101285; doi:10.3389/fendo.2021.565714)
Supplement: Supplementary file 3 [file Table_1.docx]

**Supplementary Table 1.** Baseline Characteristics of PA and non-PA patients.

| Variables | PA | Non-PA | *P* |
| --- | --- | --- | --- |
| n (male/female) | 95/127 | 95/127 |  |
| Age (year) | 51±11 | 52±12 | 0.975 |
| Body mass index (kg/m^2^) | 24.7±3.7 | 23.7±3.5 | 0.764 |
| Systolic blood pressure | 144±20 | 123±15 | < 0.05 |
| Diastolic blood pressure | 90±14 | 76±10 | < 0.01 |
| Duration of hypertension (years) | 8±7 | 7±6 | 0.57 |
| Antihypertensive medication (n/%) | 40/18% | 38/17% | 0.532 |
| Diuretics | 0/0% | 0/0% |  |
| Calcium antagonists | 24/11% | 22/10% |  |
| Beta blockers | 6/3% | 7/3% |  |
| ACEIs or ARBs | 10/4% | 9/4% |  |
| NAFLD (n/%) | 78 (35.1%) | 66 (29.7%) | < 0.05 |
| Potassium (mmol/L) | 3.44±0.63 | 3.95±0.33 | < 0.05 |
| Potassium supplementation (%) | 60% |  |  |
| Pathological type (n/%) |  |  |  |
| Aldosteronoma | 45/20% |  |  |
| Hyperplasia | 150/68% |  |  |
| Laterality (n/%) |  |  |  |
| Left | 98/44% |  |  |
| Right | 90/41% |  |  |
| Bilateral | 23/10% |  |  |
| Aldosterone (pg/mL) | 262±170 |  |  |

NAFLD, non-alcoholic fatty liver disease; ACEI, angiotensin-converting enzyme inhibitors; ARB, angiotensin receptor blockers.
