# Supplementary material for: Non-Alcoholic Fatty Liver Disease and Hypokalemia in Primary Aldosteronism Among Chinese Population
Source: Front Endocrinol (Lausanne). 2021 Apr 22;12:565714. doi: 10.3389/fendo.2021.565714 (PMC8101285; doi:10.3389/fendo.2021.565714)
Supplement: Supplementary file 4 [file Table_2.docx]

**Supplementary Table 2.** Metabolic and inflammatory state in normokalemia and supplemented hypokalemia patients with primary aldosteronism.

| Variables | Normokalemia | Supplemented Hypokalemia | *P* |
| --- | --- | --- | --- |
| n | 111 | 111 |  |
| Aldosterone (pg/mL) | 232±130 | 317±220 | < 0.05 |
| Potassium (mmol/L) | 3.89±0.20 | 3.27±0.68 | < 0.05 |
| Potassium supplementation | 0/0% | 105/95% | < 0.001 |
| Post-treatment Potassium (mmol/L) | 3.87±0.24 | 3.68±0.38 | 0.402 |
| PRA (ng/mL/h) | 0.459±0.178 | 0.223±0.125 | < 0.05 |
| Urinary aldosterone (μg/24 h) | 6.07±4.54 | 8.75±6.16 | < 0.05 |
| Urine potassium (mmol/24 h) | 43.5±25.1 | 53.3±30.3 | 0.107 |
| Body mass index (kg/m^2^) | 23.8±4.9 | 25.3±3.8 | < 0.05 |
| Systolic blood pressure | 146±23 | 149±25 | 0.675 |
| Diastolic blood pressure | 92±17 | 93±19 | 0.709 |
| Total bilirubin (μmol/L) | 10.7±5.6 | 12.0±5.9 | 0.109 |
| Triglyceride (mmol/L) | 1.43±0.87 | 1.69±0.76 | < 0.05 |
| Total cholesterol (mmol/L) | 4.13±0.75 | 4.38±0.80 | < 0.05 |
| HDL-cholesterol (mmol/L) | 1.16±0.30 | 1.08±0.29 | 0.123 |
| LDL-cholesterol (mmol/L) | 2.25±0.64 | 2.37±0.65 | 0.223 |
| VLDL-cholesterol (mmol/L) | 0.77±0.50 | 0.84±0.35 | 0.209 |
| Fasting plasma glucose (mmol/L) | 4.80±1.12 | 5.01±1.54 | 0.378 |
| Hb1Ac (%) | 6.11±1.32 | 6.14±1.25 | 0.953 |
| HOMA-IR | 1.60±1.27 | 2.01±1.84 | 0.069 |
| Serum uric acid (μmol/L) | 317±89 | 325±105 | 0.073 |
| CRP (mg/L) | 2.3±1.7 | 2.7±2.5 | 0.241 |
| WBC (*10^9) | 5.9±1.8 | 6.3±2.2 | 0.108 |
| Neutrophil (*10^9) | 3.6±1.5 | 4.3±2.3 | 0.237 |
| PLR | 88±24 | 100±45 | 0.058 |

HDL-cholesterol, high-density-lipoprotein cholesterol; LDL-cholesterol, low-density-lipoprotein cholesterol; VLDL-cholesterol, very-low-density-lipoprotein cholesterol; PRA, plasma renin activity; WBC, white blood count; CRP, C-reactive protein; PLR, platelet-to-lymphocyte ratio.
